# Silicone-Induced Granuloma after Injection for Cosmetic Purposes: A Rare Entity of Calcitriol-Mediated Hypercalcemia

**DOI:** 10.1155/2013/807292

**Published:** 2013-12-09

**Authors:** Nidhi Agrawal, Sinan Altiner, Nicholas H. E. Mezitis, Sina Helbig

**Affiliations:** ^1^Columbia University College of Physicians and Surgeons, 630 West 168th Street, New York, NY 10032, USA; ^2^St. Luke's Roosevelt Hospital Center, Division of Endocrinology, 1111 Amsterdam Avenue, New York, NY 10025, USA; ^3^St. Luke's Roosevelt Hospital Center, 1111 Amsterdam Avenue, New York, NY 10025, USA; ^4^St. Luke's Roosevelt Hospital Spencer Cox Center for Health, 1000 10th Avenue, New York, NY 10019, USA; ^5^St. Luke's Roosevelt Hospital Center, Division of Infectious Diseases, 1111 Amsterdam Avenue, New York, NY 10025, USA

## Abstract

Hypercalcemia is often a clue to the presence of unsuspected illness. We present an interesting case of an HIV-positive transgender female with a rare cause of silicone-induced granulomatous hypercalcemia. Although there have been a few case reports of silicone injections in dialysis patients causing hypercalcemia, this metabolic derangement secondary to silicone granulomas continues to be a unique entity with an unclear pathophysiology. We present a 45-year-old transgender HIV-positive female, with extensive silicone injections, who presented with symptomatic hypercalcemia. Workup for malignancy and hyperparathyroidism was negative. 1,25-Dihydroxyvitamin D level and 24-hour urine calcium level were elevated. CT scan showed extensive high-density reticulonodular densities in the buttocks and gluteal muscle fascia extending upwards to the lumbar region, along with prominent external iliac and inguinal lymph nodes. Nuclear imaging showed diffuse heterogeneity and increased uptake in the buttocks, most consistent with granuloma calcifications, and an inguinal lymph node biopsy confirmed a foreign body giant cell reaction. The patient was started on prednisone and this resulted in decrease in serum and urinary calcium levels. Physicians should have a high index of suspicion for silicone-induced hypercalcemia considering the growing prevalence of body contour enhancement with injections, implants, and fillers using this material.

## 1. Introduction

Hypercalcemia is often a clue to unsuspected illness. If hyperparathyroidism and malignancy are ruled out, rare causes of hypercalcemia, which account for less than 10% of cases of elevated serum calcium, need to be entertained. Hypercalcemia mediated by 1,25-dihydroxyvitamin D (calcitriol) is uncommon, with evidence on etiology limited to small case series or reports. We report a case of silicone granuloma-induced hypercalcemia in an HIV-positive transsexual male to female. This case adds to the scarce literature of this entity, the pathogenesis of which is incompletely understood. In light of the growing trend of silicone injection practices for cosmetic purposes around the world, this case highlights a complex complication.

## 2. Case Report

A 45-year-old transsexual male to female presented with complaints of fatigue and abdominal pain. Laboratory studies revealed hypercalcemia and renal failure. Her medical history included well-controlled human immunodeficiency virus (HIV) infection (CD4 581 cells/mm^3^, viral load <50 copies/mL), chronic anemia, and depression. She reported daily compliance to highly active antiretroviral therapy including tenofovir, emtricitabine, elvitegravir, and cobicistat as well as hormone therapy with monthly estrogen injections, daily finasteride, spironolactone, and fluconazole. She denied the use of vitamin A or D and over-the-counter calcium-containing supplements. Upon further investigation, she volunteered a history of multiple silicone injections to the buttocks and cheeks fifteen years ago at a clinic in Latin America, as well as silicone implants to both breasts two years ago.

The review of systems was negative for constipation, change in urination, and bone pain. Physical examination revealed a phenotypically normal-appearing Hispanic female with unremarkable vital signs. Her abdomen was mildly diffuse tender. There were no signs of redness, tenderness, or swelling over the breasts or buttocks. She had bilateral palpable inguinal lymphadenopathy in the absence of cervical and axillary lymphadenopathy. Laboratory results were significant for total calcium of 13.1 mg/dL (normal range 8.4–10.3 mg/dL), ionized calcium of 6.9 mg/dL (normal range 4.8–5.6 mg/dL), albumin of 3.8 mg/dL, along with a creatinine of 3.0 mg/dL, and phosphorus of 3.6 mg/dL (normal range 2.5–4.5 mg/dL).

A detailed workup for new-onset hypercalcemia was performed. The parathyroid hormone (PTH) level was suppressed <3 pg/mL (normal range 11–67 pg/mL) and the PTH-related protein was normal at 17 pg/mL (normal range 14–27 pg/mL) as were thyroid stimulating hormone (TSH) and cortisol levels. Serum levels of 25-hydroxy vitamin D3 were 17 pg/mL and 1,25-dihydroxyvitamin D3 were elevated at 147 pg/mL (normal range 18–72 pg/mL).

**Figure 1:**
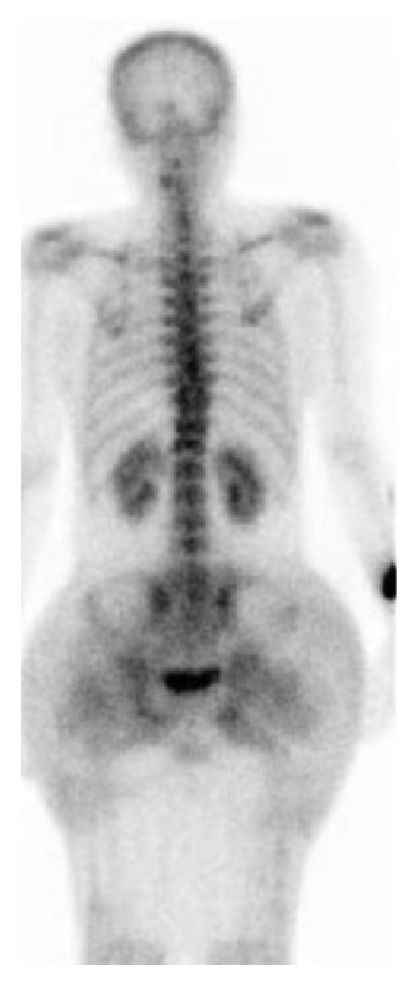
Tc labeled bone scan revealing diffuse heterogeneity and increased uptake at the bilateral buttocks.

A noncontrast computer tomography (CT) of the chest, abdomen, and pelvis revealed no evidence of a malignant focus. There were, however, extensive high-density reticulonodular densities in the buttocks and gluteal muscles extending to the lower back consistent with body sculpting silicone injections along with prominent external iliac and inguinal nodes. A technetium-labeled whole body scan showed diffuse heterogeneity and increased uptake at the bilateral buttocks suggestive of granuloma calcification related to silicone injection (Figure 1).

**Figure 2:**
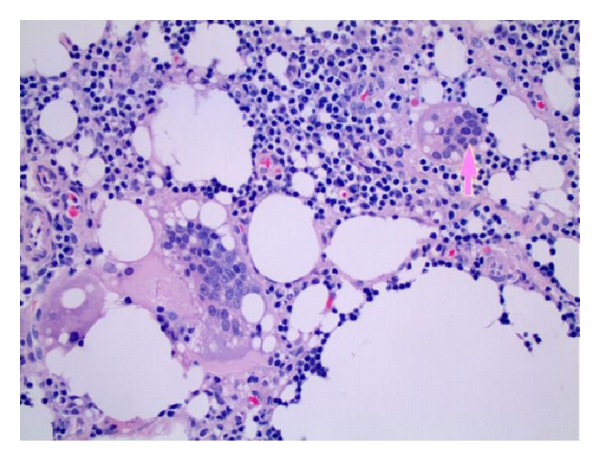
Inguinal lymph node excisional biopsy showing giant cells (arrow).

An inguinal lymph node biopsy showed changes consistent with sinus histiocytosis and foreign body giant cell reaction, suggesting foreign body induced granuloma formation secondary to the gluteal injections (Figure 2).

Serum angiotensin converting enzyme (ACE) level was 117 U/L (normal range 9–67 U/L); however in the absence of clinical and computed tomographic findings suggesting sarcoidosis in addition to normal pulmonary function tests and an unremarkable ophthalmologic examination, sarcoidosis was deemed unlikely to be the culprit. Additional malignancy workup, consisting of prostate specific antigen, serum- and urine protein electrophoresis were negative.

The serum calcium improved to 11.3 mg/dL with hydration and a calcium restricted diet in 3 days. The creatinine level stabilized at 1.8 mg/dL. A 24 hr urine collection showed a calcium level of 613 mg/24 hrs (normal range 100–321 mg/24 hrs).

Surgical removal of the silicone deposits and reactive tissue overlying her hips were not considered an option. The patient refused for cosmetic purposes. The surgical consultants deemed excision unsafe and ineffective due to the extent of injections with partial migration.

The patient was started on oral daily prednisone 30 mg and a calcium restricted diet. Calcium levels gradually returned to normal. Repeat 24-hour urine calcium improved to 580 mg/24 hours and serum levels of 1,25-dihydroxyvitamin D3 normalized to 56 pg/mL after two days of steroid treatment. A gradual taper of prednisone was initiated after 2 weeks of treatment and calcium levels and renal function remained stable. A repeat 24 hr urine calcium 3 weeks after starting prednisone had decreased to 198 mg/24 hours.

## 3. Discussion

Hypercalcemia is often a clue to unsuspected illness. If hyperparathyroidism and malignancy are ruled out, rare causes of hypercalcemia, which account for less than 10% of cases of elevated serum calcium, need to be entertained. We report a case of silicone granuloma-induced hypercalcemia in an HIV-positive transsexual male to female.

Silicone in the form of polymethyl siloxane is a popular injectable soft-tissue filler. Its chemical inertness, low surface tension, and resistance to composition by heat have contributed to the widespread use of silicone as injectable cosmetic material [1]. Although these characteristics enable liquid silicone to achieve excellent results in soft-tissue augmentation, improper injection practices may lead to systemic complications, disfigurement, and migration of the silicone [2–4].

Frequently, surgical removal of the silicone is not possible, resulting in a lifelong negative impact on quality of life. Winer and colleagues [1] first reported the occurrence of silicone granuloma in 1964. The incidence of granuloma formation in patients injected with medical grade silicone can be as high as 20 percent of patients receiving injections [3]. Occurrence of granulomatous reactions has been reported anytime from 3 weeks to 20 years after injection [4]. The exact mechanism of granuloma formation in response to silicone implantation has not been fully elucidated. T-cell activation triggered by infection, trauma, adulterants added to the silicone, or denatured host proteins such as fibrinogen adsorbed to the silicone has been proposed as the initial pathomechanism [4–6]. Once activated, T-cells release cytokines, including tumor necrosis factor alpha, which promote granuloma formation. Although granulomas represent an adverse effect of silicone injections independent of the purity of the silicone used, they have rarely been considered as a cause of hypercalcemia.

An extensive search of the literature revealed three reported cases of hypercalcemia secondary to silicone injections for cosmetic reasons [7–9]. Silicone-induced hypercalcemia has also been reported in two dialysis patients secondary to the silicone content of their dialysis tubing, which resolved when these lines were replaced by silicone-free material [10].

Silicone granuloma-induced hypercalcemia is linked to increased levels of plasma calcitriol (1,25-dihydroxyvitamin D3). Hypercalcemia associated with increased levels of plasma calcitriol is a common finding in granulomatous diseases such as sarcoidosis and tuberculosis, as well as in certain systemic fungal infections. Macrophages residing in the granulomas convert 25-hydroxy vitamin D to 1,25-dihydroxyvitamin D3 with the help of the 1*α*-hydroxylase, leading to hypercalcemia by increase of gut calcium absorption and calcium mobilization from bone [11].

HIV infection can be associated with hypercalcemia, usually in the setting of poor viral control. It may occur in the setting of HIV-associated lymphadenopathy and immune reconstitution syndrome. AIDS-related *Mycobacterium avium-intracellulare* infection and diffuse cytomegalovirus infection can precipitate elevated serum calcium levels. The hypercalcemia in these settings is not known to be calcitriol-mediated hypercalcemia [11].

Our patient had a classical presentation of calcitriol-mediated hypercalcemia with hypercalciuria, elevated levels of serum calcitriol, and suppressed levels of PTH, accompanied by a negative workup for malignancy and sarcoidosis. Cases in the literature report biopsy results of the silicone-augmented areas to confirm the granulomatous reaction. In our case a regional lymph node biopsy without biopsy of the soft tissue confirmed the diagnosis.

Physicians should have a high index of suspicion for silicone-induced hypercalcemia considering the growing prevalence of body contour enhancement with injections, implants, and fillers using this material.
